# Advantage of Handwriting Over Typing on Learning Words: Evidence From an N400 Event-Related Potential Index

**DOI:** 10.3389/fnhum.2021.679191

**Published:** 2021-06-10

**Authors:** Aya S. Ihara, Kae Nakajima, Akiyuki Kake, Kizuku Ishimaru, Kiyoyuki Osugi, Yasushi Naruse

**Affiliations:** ^1^National Institute of Information and Communications Technology, and Osaka University, Kobe, Japan; ^2^Wacom Co., Ltd., Kazo, Japan; ^3^Graduate School of Frontier Bioscience, Osaka University, Suita, Japan

**Keywords:** handwriting, typing, digital device, learning, electroencephalography, digital pen, mood, N400

## Abstract

The growing implementation of digital education comes with an increased need to understand the impact of digital tools on learning. Previous behavioral studies have shown that handwriting on paper is more effective for learning than typing on a keyboard. However, the impact of writing with a digital pen on a tablet remains to be clarified. In the present study, we compared learning by handwriting with an ink pen on paper, handwriting with a digital pen on a tablet, and typing on a keyboard. Behavioral and electroencephalographic indices were measured immediately after learning with each writing tool. The moods of the subjects during the training were also assessed. The participants were divided according to their use of digital pen in their everyday lives, allowing us to take into account the effect of the familiarity with the digital pen on the learning process (familiar group vs. unfamiliar group). We performed an EEG experiment applying a repetition priming paradigm. In each trial, a learned foreign language word (prime word) and a mother tongue word (target word) were consecutively presented. The target word was either semantically identical to the prime word (repetitive condition) or different (non-repetitive condition). We assumed that a larger priming effect on N400 reflects larger learning progress. The familiar group showed a greater N400 priming effect for words learned with the digital or ink pen than those learned with the keyboard. The unfamiliar group showed the greater N400 priming effect for words learned with the ink pen compared with words learned by typing. In addition, positive mood during learning was significantly higher during handwriting than during typing, regardless of the groups. On the other hand, the behavioral indices were not influenced by the writing tool. These results suggest that the movements involved in handwriting allow a greater memorization of new words. The advantage of handwriting over typing might also be caused by a more positive mood during learning. Finally, our results show that handwriting with a digital pen and tablet can increase the ability to learn compared with keyboard typing once the individuals are accustomed to it.

## Introduction

To develop efficient education systems, assessing the effect of the use of digital tools on learning is essential. Regarding writing tools, previous behavioral studies have shown that handwriting is more effective for learning than keyboard writing. For example, Longcamp et al. (2005) showed a better recognition of the alphabet letters in preschool children after handwriting training compared with typing training. Similar results were reported in adults for the learning of pseudo-letters (Longcamp et al., 2006). In addition, preschool children who practiced handwriting of the alphabet performed better in free letter and word writing than those who underwent typing training (Kiefer et al., 2015). In adults, free word recall was better for words that were written by hand rather than typed on both a conventional or a touch keyboard (Mangen et al., 2015). The advantage of handwriting over typing is considered to originate from the meaningful coupling between action and perception (Kiefer et al., 2015), on the basis of evidence from electroencephalography (EEG) (van der Meer and van der Weel, 2017) and magnetic resonance imaging studies (Longcamp et al., 2008; Vinci-booher et al., 2016). Finally, a behavioral study showed that university students who took notes on a laptop performed worse on conceptual questions than those taking notes by hand. This detrimental effect of typing was suggested to lay in the fact that typing is a verbatim transcription of the lecture, while writing by hand requests processing and rephrasing of the information (Mueller and Oppenheimer, 2014).

These studies focused on conventional handwriting, namely, writing with a pen or pencil on paper. A few reports have investigated the impact of writing with a digital pen on a tablet on learning. Hatano et al. (2015) recorded EEG signals while high school students attending science class were taking notes with a digital pen on a tablet and with a pencil on paper. They found a higher theta-frequency activity in the frontal area of the brain from students taking notes on the tablet compared with those writing on paper. However, the scores achieved at the comprehension and memory tests conducted after the note-taking did show no difference. The study proposed that using the tablet required enhanced cognitive effort to monitor the writing at the cost of content processing and learning. A clear disadvantage of the use of digital tools for handwriting was recently shown in a behavioral study. Preschool children learning the alphabet during 7 weeks were divided into three groups: (1) children writing with a pencil on paper, (2) children writing with a stylus on a tablet, and (3) children typing with a virtual keyboard on a tablet (Mayer et al., 2020). The group using a pencil and paper, but not the group using the stylus and tablet, performed better on letter recognition and showed improved visuospatial skills compared with the group using a virtual keyboard. In addition, the children typing on the keyboard achieved better word writing and reading than those using the stylus and tablet. Thus, writing with a stylus on a tablet might be a less effective way to acquire literacy, possibly because of the increasing demand for motor control. Indeed, several kinematics studies showed impaired motor control when writing with a digital pen on a tablet. For example, a disturbance in the segment trajectory calculation and a reduced control of muscular adjustment were observed (Alamargot and Morin, 2015; Gerth et al., 2016a,b; Wollscheid et al., 2016; Guilbert et al., 2019).

However, writing with a digital pen might be disadvantageous as it is an unfamiliar tool. In a previous study (Osugi et al., 2019), we used N400, an event-related potential (ERP) response to compare the effect of handwriting tools (ink pen vs. digital pen) on learning. Then, we took into account the familiarity of the subjects with digital pens as we divided the participants according to their use of digital pen and tablet in their everyday lives (familiar vs. unfamiliar). N400 is related to semantic processing that is elicited by various kinds of stimuli, such as words, speech, and pictures (for a review, see Kutas and Federmeier, 2011). The amplitude of N400 is modulated by the ease of accessing information from long term memory and integrating semantic representations into a preceding context (for a review, see Kutas and Federmeier, 2000). Thus, N400 changes during language learning (Ojima et al., 2005, 2011) and developmental progress (Friedrich and Friederici, 2004, 2010; Reid et al., 2009). One important characteristic is that the change of N400 occurs in an earlier stage of learning compared with behavioral responses (McLaughlin et al., 2004). Therefore, N400 is a powerful tool to reveal the effects of learning, especially in the early stage. In our previous study, in the familiar group, the greater N400 effect was recorded for words learned by writing with a digital pen on a tablet, while no significant difference between the words was found in the unfamiliar group. This suggests that, after individuals become accustomed to writing with a digital pen on a tablet, it might be an effective writing tool with regard to learning.

In order to further clarify the effects of the writing tool on brain activity after learning, we conducted an ERP study which builds on our previous study (Osugi et al., 2019) in the following ways: (1) typing was included as an additional writing method, (2) mood assessment was conducted using the profile of mood states (POMS) questionnaire, and (3) participants were given words in an unknown language words (Indonesian) to learn, instead of difficult words in their mother tongue (Japanese), as in the previous paper. To account for the potential effects of familiarity with digital pens, we divided participants into two groups according to whether they routinely used digital pens. As in the previous study, we used the N400 response as an index of learning effect. We recorded EEG signals from adult participants who were native Japanese speakers after they learned Indonesian words by either handwriting with an ink pen on paper, handwriting with a digital pen on a tablet, or typing with a keyboard on a laptop. In our previous study (Osugi et al., 2019), most participants who were familiar with digital pens felt that writing with an ink pen required more effort, while the unfamiliar group perceived writing with a digital pen to be more demanding. Furthermore, most participants who were accustomed to digital pens enjoyed writing with one more than with an ink pen, while a slight majority of the unfamiliar participants favored an ink pen. Therefore, we also investigated whether the mood of the participant during learning was affected by the writing tool used, based on a quantitative analysis.

## Materials and Methods

### Participants

We recruited right-handed native Japanese speakers with normal hearing and normal/corrected-to-normal vision. The participants had no history of psychiatric disease and did not speak nor were exposed to the Indonesian language. Additional criteria were as followed: all participants regularly used a keyboard in their everyday lives (more than a few days); participants who wrote with a digital pen on a tablet in their everyday lives (more than a few days) constituted the familiar group and those who did not use it at all formed the unfamiliar group. In total, 39 participants (familiar group, 12 participants; unfamiliar group, 27 participants) were recruited for the experiments. Data measured from six participants who showed an average task accuracy in ERP experiments of less than 60% were excluded from the study. Therefore, we analyzed the data obtained from 33 participants (8 women; age range, 21–48 years old) distributed as follows: 12 participants (2 women; average age, 37.5 ± 5.9 years) for the familiar group and 21 participants (6 women; average age, 34.4 ± 10.0 years) for the unfamiliar group. The study protocol was approved by the Bioinformatics Ethics Committee of the National Institute of Information and Communications Technology, and all subjects provided written informed consent before participating in this study.

### Learning Materials

#### Selection of Words for the Learning Activity

We selected 60 Indonesian common words, including animal names (e.g., tiger, sheep), body parts (e.g., leg, mouth), and person (e.g., mother, teacher), all of which contained 3–5 letters (Supplementary Table 1). Japanese translations of the Indonesian words were high-frequency words (common logarithm value of frequency per million: 1.7 ± 0.5^1^) with high familiarity for Japanese people (6.3 ± 0.4 on a 7-point scale) (Amano and Kondo, 1999) and were written in 1–3 kanji morphogram(s) and/or kana syllabogram(s) with 1–4 morae. Sixty pairs of the Indonesian word and the corresponding Japanese word were divided into three learning sets of 20 pairs each. We confirmed that the lexical properties of the Japanese words were matched among the sets based on the results of the Kruskal-Wallis test. Indeed, there were no significant differences in the frequency (*p* = 0.25) or in the familiarity (*p* = 0.25) across the sets (Table 1). In the learning activity, each participant learned these three sets of words that were randomly selected across the participants.

**TABLE 1:** Lexical properties of the Japanese words corresponding to the Indonesian words in three learning sets.

Lexical property	Set 1	Set 2	Set 3	Kruskal-Wallis test
Common logarithm value of frequency per million	1.6 ± 0.5	1.5 ± 0.7	1.8 ± 0.4	n.s.
Familiarity value (7 grades)	6.4 ± 0.2	6.2 ± 0.4	6.2 ± 0.4	n.s.

#### Stimuli for ERP Experiment

**FIGURE 1:**
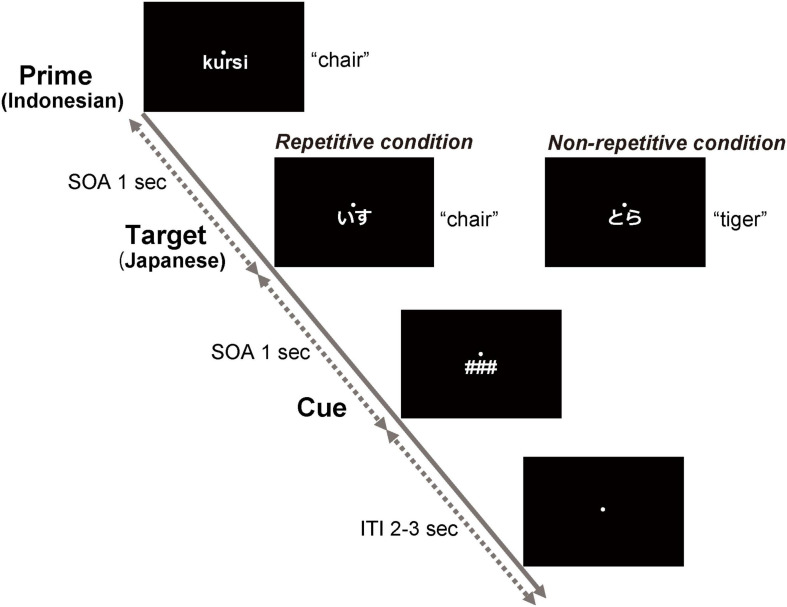
Schematic representation of the repetition priming paradigm. The prime stimuli were Indonesian words written in alphabet, which the participants wrote in the learning activity. The target stimuli were Japanese words written in Japanese morphograms (kanji) and/or syllabograms (kana). The target words were Japanese translations of the prime stimuli in the repetitive condition (i.e., semantically repetitive). In the non-repetitive condition, the target words were not related to the prime stimuli. The prime, target, and cue were presented with a stimulus-onset asynchrony of 1 s. The intertrial interval between the offset of the cue and onset of the next prime was randomly set at 2–3 s. After the presentation of the cue (###), the participants answered whether the target word matched the prime word or not by clicking on a mouse with the right fingers.

For the ERP experiment, we used a repetition priming paradigm in which the Indonesian words were presented (prime words) and then followed by the Japanese words (target words). The prime and target words were semantically identical (repetitive condition) or not (non-repetitive condition) (Figure 1). The word pairs in the non-repetitive condition were constituted of an Indonesian word from one set and a Japanese word from another set. To prevent unwanted influences from phonological and semantic priming effects on the non-repetitive condition, two evaluators checked the presence of phonological similarity and semantic relationships between the prime and target words in each pair. Only the word pairs approved by both evaluators were used. Each Indonesian word was presented four times as the prime stimulus throughout the experiment: twice for the repetitive condition and twice for the non-repetitive condition. Hence, the participants underwent 240 trials in total, which are organized as follows: the prime stimuli were words written with an ink pen for 40 trials with the repetitive condition and 40 trials with the non-repetitive condition, the prime stimuli were the words written with a digital pen for 40 trials with the repetitive condition and 40 trials with the non-repetitive condition, and the prime stimuli were the words written with a keyboard for 40 trials with the repetitive condition and 40 trials with the non-repetitive condition.

### Experimental Procedures

**FIGURE 2:**
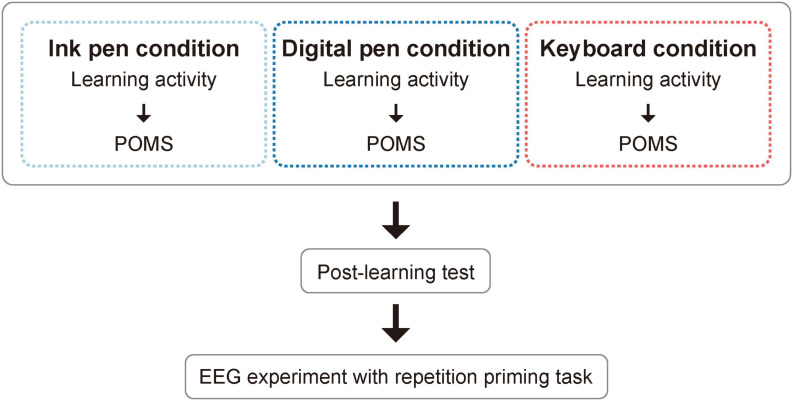
Flowchart showing the study design.

The experimental flow was as follows (Figure 2): (1) learning activity and rating of mood states, (2) post-learning test, and (3) EEG measurement. This was all conducted at the same session. The protocols for each of these steps are described below.

#### Learning Activity

We used a counterbalanced design to determine the order in which participants received each of the following three conditions in the learning activity: the “ink pen” condition, in which the participants wrote the Indonesian words with an ink pen on paper; the “digital pen” condition, in which they wrote with a digital pen on a tablet; and the “keyboard” condition, in which they typed with a keyboard. The participants were given learning sheets on which 20 Indonesian words and their corresponding Japanese translations were written (Supplementary Figure 1). In each condition, each sheet contained the same 20 pairs of words and participants were asked to write the 20 Indonesian words repeatedly on the learning sheets to try to memorize them. As soon as the participants finished one sheet, they continued this process on the next sheet, until the 10 min time limit was reached.

Twenty pairs of Indonesian words and the corresponding Japanese words were written on each learning sheet. The participants were asked to write the Indonesian words to memorize. For the ink pen condition, the learning sheet was physically placed on a pen tablet (Intuos Pro Large PTH-851; Wacom Co., Ltd.), and the participants were instructed to write with an ink pen (Ink Pen KP-130-01; Wacom Co., Ltd.). For the digital pen condition, a PDF file of the learning sheet was displayed to the participants on a pen display (Cintiq 13HD Creative Pen Display DTK-1301; Wacom Co., Ltd.) while the participants wrote with a digital pen (Pro Pen KP503E; Wacom Co., Ltd.). For the keyboard condition, a PDF file of the learning sheet was displayed to the participants on a PC display (ColorEdge CX241; EIZO Corporation) while the participants typed with a keyboard (USB Wired Keyboard 104 Keys Black and Silver KU-0316; HP Inc.).

#### Rating of Mood States During Learning

To assess the mood states of the participants during each learning activity, we used the POMS2 (2nd edition), short version, for Japanese adults (Heuchert and McNair, 2015), which measures the following dimensions of mood: *Anger or Hostility*, *Confusion or Bewilderment*, *Depression or Dejection*, *Fatigue or Inertia*, *Tension or Anxiety*, and *Vigor or Activity*. At the end of each learning activity, the participants assessed their mood during the learning activity on a 5-point scale (0, not at all; 4, extremely). This rating was completed within 5 min.

#### Post-learning Test

The performance in memorizing the Indonesian words was tested after the learning activities. The test sheet given to the participants contained all of the Indonesian words, and the participants answered by writing the Japanese translations.

#### EEG Measurement

The prime, target, and cue (###) were visually presented continuously with each stimulus-onset asynchrony set to 1,000 ms (Figure 1). The presentation duration for the prime and target stimuli was 300 ms, and for the cue, it was 500 ms. The participants were asked to silently read the prime (Indonesian word) and target (Japanese word) and answer whether they matched or not by clicking on the computer mouse with the right hand after the presentation of the cue. The prime for the next trial was presented 2,000–3,000 ms after the cue onset. We used MATLAB (MathWorks, Inc.) and the Psychophysics Toolbox Version 3^2^ to run the repetition priming task.

EEG and electrooculography (EOG) signals were continuously measured using an eight-channel wearable EEG device (PolymateMini AP108; Miyuki Giken Co., Ltd., Tokyo, Japan). The dry midline electrodes (Unique Medical Co., Ltd., Tokyo, Japan) were placed at Fz, Cz, and Pz according to the International 10–20 system. In addition, an electrode was placed on the upper and right sides of the left eye to measure the vertical and horizontal EOG components. The EOG recording allowed the detection of the artifactual eye movements and blinks and the removal of the noise components from the EEG signals. All signals were sampled at 500 Hz with the use of the left earlobe as the ground and the right earlobe as the reference.

### Data Analyses

#### Behavioral Indices

We recorded the number of words written in 10-min learning activity, the number of correct answers on the post-learning test, and the accuracy rates for the judgment task in the EEG experiment for each participant and each learning set. To assess the differences in these behavioral indices between groups and writing tools, a two-factor mixed analysis of variance (ANOVA) was performed with participant groups (familiar and unfamiliar) and writing tools (ink pen, digital pen, and keyboard) as factors. The significance level was set at 5%. If the Mauchly’s test showed that homogeneity of variance was violated, the degree of freedom was adjusted using the Huynh-Feldt procedure. When a significant interaction was obtained, one-way repeated measures ANOVA was performed for each group, and an unpaired *t*-test was performed for each writing tool. For all multiple comparisons, *p*-values were adjusted using the Benjamini-Hochberg procedure. Statistical analyses in the present study were conducted using IBM SPSS statistics 24.0J software (IBM).

#### Mood Indices

For each participant, we converted the raw scores on each of the six dimensions to a standardized score (*T*-score), using the raw score to *T*-score conversion tables from the POMS 2 manual of POMS 2 in which the *T*-scores were calculated using the mean and standard deviation from a sample of Japanese adults (*n* = 2,787) (Heuchert and McNair, 2015). A two-factor mixed ANOVA was then performed to analyze the POMS standardized scores for each mood dimension as described for the analysis of the behavioral indices.

#### Electroencephalographic Indices

The analysis of the EEG and EOG signals was conducted using MATLAB (MathWorks, Inc.) and the EEGLAB toolbox (Delorme and Makeig, 2004). A FIR band-pass filter of 0.5–20 Hz (3,000th) was applied to the measured EEG and EOG signals. The artifact components, mainly caused by eye movements and blinking, were excluded from the EEG signals using noise reduction processing with artifact subspace reconstruction and independent component analysis. In addition, we excluded from the average any trial exceeding ±30 μV on the Fz, Cz, and Pz channels or exceeding ±100 μV on the vertical and horizontal EOG channels. Next, the signals from 100 ms before to 800 ms after target onset were averaged for each condition (repetitive vs. non-repetitive) and each channel. The signals were then corrected using 100 ms before target onset as a baseline.

In this study, difference in N400 amplitude between the repetitive and non-repetitive conditions (i.e., repetition priming effect on N400) was used as an indicator of learning: we assumed that, as learning progressed, a larger difference would occur. To detect a repetition priming effect on N400, the ERP for the repetitive condition was subtracted from the ERP for the non-repetitive condition. A large repetition priming effect (i.e., difference between repetitive and non-repetitive) was shown at the Cz electrode location. Therefore, we averaged the amplitudes of the differential EEG responses at Cz from 300 to 450 ms after target onset and used them as electroencephalographic indices to assess the learning effect of each writing tool. A two-factor mixed ANOVA was used to analyze the difference of the N400 priming effect between the groups and between the writing tools as described above (paragraph 2.4.1.).

#### Comparison With the Results of Our Previous Study

We compared the learning effect of handwriting with the digital pen and with an ink pen acquired in this study with those obtained in our previous study (Osugi et al., 2019). Previously, the participants were also divided into two groups: those who used the digital pen in their daily lives for the familiar group (*N* = 11) and those who did not for the unfamiliar group (*N* = 17). Five of them participated in the present study as well. In our 2019 work, the participants learned to read difficult words of their mother tongue by handwriting with an ink and a digital pen. We then conducted an EEG experiment with a repetition priming paradigm similar to what was done here. Briefly, the words written either with an ink or a digital pen in the learning period (prime words) were followed by the words that were reading representation of the prime words (repetitive condition) or not (non-repetitive condition). The learning time, number of words to learn with each pen type, time sequence, and judgment task of the priming paradigm were identical to those used in the present study. Using the behavioral (i.e., numbers of correct answers on the post-learning test and accuracy rates for the judgment task in the ERP experiment) and ERP (i.e., N400 priming effect) indices extracted in the present and 2019 studies, a two-factor ANOVA for each participant group was performed with experiment (previous study and present study) and writing tool (ink pen and digital pen) as factors. The between-experiment difference for each writing tool was assessed using an unpaired *t*-test.

## Results

### Behavioral Indices

**FIGURE 3:**
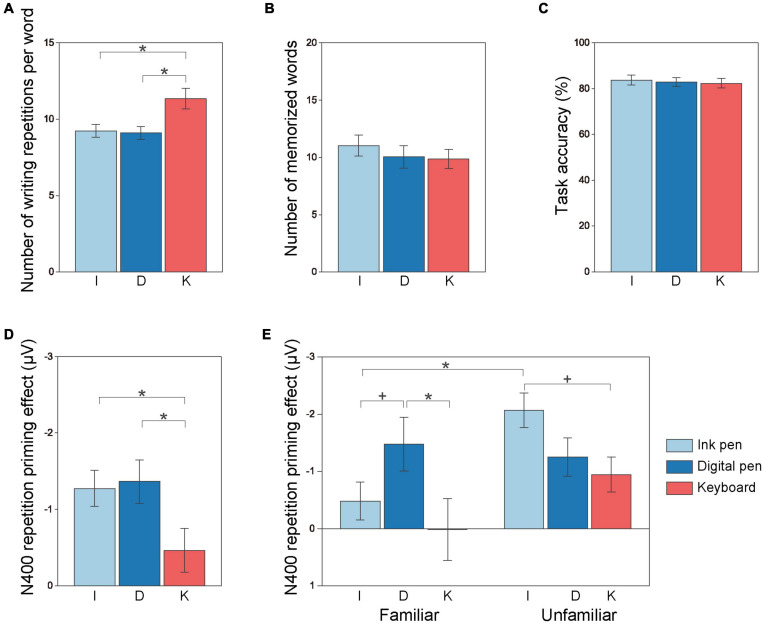
Learning effect of the three methods on behavioral and electroencephalographic indices. The number of writing repetitions per word during the learning activity **(A)** was significantly greater when written with a keyboard (red) compared with when handwritten with an ink pen (light blue) or a digital pen (dark blue), regardless of the group. The numbers of memorized words in the posttest **(B)** and task accuracy in the EEG experiment **(C)** were not affected by the writing tool. The repetition priming effect on N400 was greater for words learned using an ink pen or a digital pen than for words learned using a keyboard **(D)**. In addition, a significant interaction between the group and writing tool was found **(E)**. In the familiar group, the repetition priming effect was significantly greater for words learned with a digital pen than for those learned with a keyboard. In the unfamiliar group, the repetition effect was marginally greater for words learned with an ink pen than for those learned with a keyboard. Each bar shows the grand average of the participants. The error bar represents the standard error. *p < 0.05; ^+^*p* = 0.06.

The numbers of writing repetitions produced by participants per word in the ink pen, digital pen, and keyboard conditions were 9.23 ± 0.90 (mean ± SD), 9.45 ± 0.66, and 12.12 ± 0.98, respectively, for the familiar group and 9.25 ± 0.40, 8.70 ± 0.52, and 10.55 ± 0.55, respectively, for the unfamiliar group (Supplementary Figure 2A). The statistical analysis revealed a main effect of the writing tool [*F*(1.32, 39.6) = 7.27; *p* = 0.006, partial η*^2^* = 0.20; Figure 3A]. Indeed, the number of writing repetitions was greater in the keyboard condition (11.33 ± 0.68) than in the ink pen (9.24 ± 0.43; *p* = 0.03) and digital pen (9.10 ± 0.42; *p* = 0.02) conditions (Figure 3A). No main effect of the participant group [*F*(1, 30) = 1.17; *p* = 0.29, partial η*^2^* = 0.04] and no interaction effect between the two factors [*F*(1.32, 39.6) = 0.74; *p* = 0.43, partial η*^2^* = 0.24] were detected.

In the familiar group, 10.25 ± 1.43, 9.42 ± 1.76, and 8.75 ± 0.91 words were memorized in the ink pen, digital pen, and keyboard conditions, respectively. In the unfamiliar group, the average number of words memorized was 11.81 ± 1.12, 10.67 ± 1.13, and 10.95 ± 1.15 in the ink pen, digital pen, and keyboard conditions, respectively (Supplementary Figure 2B). There was no main effect of the participant group [*F*(1, 31) = 1.01; *p* = 0.32, partial η^2^ = 0.03] or writing tool [*F*(2, 62) = 1.72; *p* = 0.19, partial η^2^ = 0.05] and no interaction effect [*F*(2, 62) = 0.25; *p* = 0.78, partial η^2^ = 0.01; Figure 3B].

In the familiar group, the accuracy rate for the judgment task was 82.5 ± 9.7 in the ink pen condition, 82.4 ± 9.7 in the digital pen condition, and 81.0 ± 9.4 in the keyboard condition. In the unfamiliar group, it was 84.9 ± 12.2, 83.1 ± 12.1, and 83.6 ± 12.6 in the ink pen, digital pen, and keyboard conditions, respectively (Supplementary Figure 2C). There was no main effect of the participant group [*F*(1, 31) = 0.27; *p* = 0.61, partial η^2^ = 0.01] and writing tool [*F*(2, 62) = 0.42; *p* = 0.66, partial η^2^ = 0.01] and no interaction effect between the group and writing tool [*F*(2, 62) = 0.22; *p* = 0.81, partial η^2^ = 0.01; Figure 3C].

### Mood Indices

**FIGURE 4:**
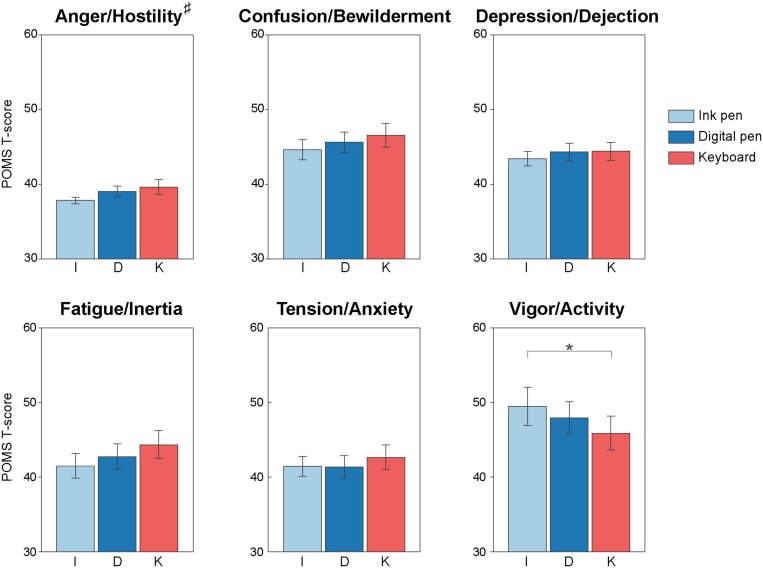
Profiles of mood states during learning with the three writing tools. The POMS *T*-scores of *Vigor or Activity* were significantly higher when learning by writing with an ink pen (light blue) than when learning with a keyboard (red). The scores of *Anger or Hostility* showed main effect on the learning (#), but *post hoc* test did not show significant differences. There was no difference between the writing tools and between the participant groups for the other negative moods, i.e., *Confusion or Bewilderment*, *Depression or Dejection*, *Fatigue or Inertia*, and *Tension or Anxiety*. Each bar shows the grand average of the participants. The error bar represents the standard error. *p < 0.05.

A positive mood state, *Vigor or Activity*, showed significant difference depending on the writing tool [*F*(2, 62) = 4.48; *p* = 0.02, partial η^2^ = 0.13; Figure 4]. The scores were significantly larger in the ink pen as compared with the keyboard condition (*p* = 0.036). A significant main effect of the writing tool was also measured for a negative mood state, *Anger or Hostilit*y [*F*(2, 62) = 3.32; *p* = 0.04, partial η^2^ = 0.10]. However, the *post hoc* analysis did not reveal significant differences between the tools. In the other mood states (i.e., *Confusion or Bewilderment*, *Depression or Dejection*, *Fatigue or Inertia*, and *Tension or Anxiety*), the writing tool had no significant main effect. There was also no significant main effect of the participant group and interaction between the group and writing tool in all mood states (Supplementary Figure 3).

### Electroencephalographic Indices: N400 Repetition Priming Effect

**FIGURE 5:**
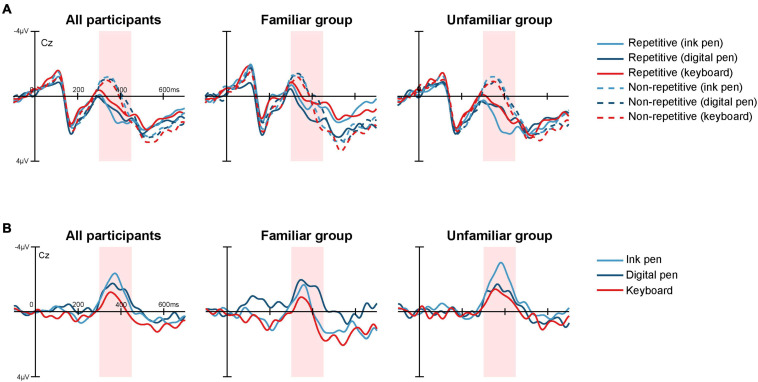
ERPs at Cz (midline vertex) measured in the repetition priming paradigm. **(A)** The ERPs for the target words learned using an ink pen (light blue), a digital pen (dark blue), and a keyboard (red) in the repetitive (solid line) and non-repetitive (dashed line) conditions were averaged across all participants (left), for the members of the familiar group (middle), and those of the unfamiliar group (right). **(B)** The ERPs for the repetitive condition were subtracted from those for the non-repetitive condition for each writing tool. The differential ERPs had a negative peak at approximately 360 ms, which represents the repetition priming effect on N400. Mean amplitudes from 300 to 450 ms (shaded in pink) were used as electroencephalographic indices for learning effect. The amplitude was higher for words learned with the ink pen (light blue) or digital pen (dark blue) than for those learned using the keyboard (red).

The ERP waveforms averaged across all 33 participants showed that the N400 peak latencies occurred later in the non-repetitive condition (ink pen, 302 ms; digital pen, 294 ms; and keyboard, 300 ms) than in the repetitive condition (ink pen, 348 ms; digital pen, 354 ms; and keyboard, 338 ms) (Figure 5A), which suggests that it took longer for semantic processing. Regardless of the writing tool, the repetitive and non-repetitive conditions showed clear differences in the grand average ERPs between 300 and 450 ms (Figure 5B). The ERP differences at Cz (i.e., non-repetitive minus repetitive) peaked around 360 ms. The amplitudes around the peaks varied depending on the writing tool, and the main effect of the writing tool was significant [*F*(2, 62) = 4.37; *p* = 0.02, partial η^2^ = 0.12]. Indeed, the repetition priming effect was greater in the ink pen (*p* = 0.04) and digital pen (*p* = 0.03) conditions than in the keyboard condition (Figure 3D). A significant main effect of the participant group was also found [*F*(1, 31) = 4.29; *p* = 0.047, partial η^2^ = 0.12] and revealed that the repetition priming effect was larger in the unfamiliar group than in the familiar group. The interaction between the group and writing tool was also significant [*F*(2, 62) = 3.72; *p* = 0.03, partial η^2^ = 0.11; Figure 3E]. In the familiar group, the repetition priming effect was significantly larger for the digital pen condition than for the keyboard condition (*p* = 0.02) (and marginally ink pen > keyboard; *p* = 0.06). In the unfamiliar group, although the between-method differences did not reach a significant level, the repetition priming effect was larger for the ink pen condition compared with the keyboard condition (*p* = 0.057). In addition, a between-group difference was shown for the ink pen condition, showing that the repetition effect was greater in the unfamiliar group than in the familiar group (*p* = 0.002). There was no between-difference for the digital pen (*p* = 0.70) and keyboard (*p* = 0.10) conditions.

### Comparison With the Results of Our Previous Study

**FIGURE 6:**
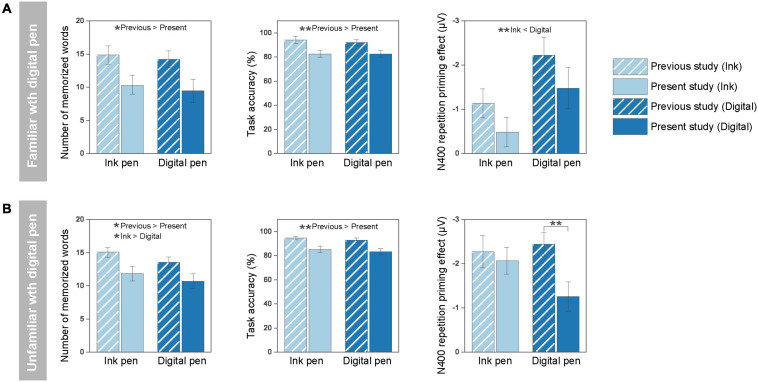
Comparison with the results of our previous study (Osugi et al., 2019): influence of the task difficulty. **(A)** In the familiar group, the number of memorized words and task accuracy were lower in the present study than in the previous one. No significant difference in the N400 priming effect was evidenced between the studies. **(B)** In the unfamiliar group, the number of memorized words and task accuracy were also lower in the present study than in the previous one. The N400 priming effect in the digital pen condition was significantly smaller in the present study than in the previous one. There was no difference in the N400 priming effect in the ink pen condition between the studies. For the number of memorized words and task accuracy, each bar shows the grand average of the two writing tools across the participants. For the N400 priming effect, each bar shows the grand average across the participants. The error bar represents the standard error. *p < 0.05, **p < 0.01. n.s., non-significant.

In the familiar group (Figure 6A), a significant main effect of the experiment was found on the number of memorized words [*F*(1, 21) = 6.00; *p* = 0.02, partial η^2^ = 0.22] and task accuracy [*F*(1, 21) = 8.83; *p* = 0.007, partial η*^2^* = 0.30]. Specifically, the performances were worse in the present study than in the previous one. There was no significant main effect of the writing tool [*F*(1, 21) = 0.70; *p* = 0.41, partial η^2^ = 0.03] and no interaction effect between the experiment and writing tool [*F*(1, 21) = 0.01; *p* = 0.91, partial η^2^ = 0.001] on the number of memorized words. Similarly, no significant main effect of the writing tool [*F*(1, 21) = 0.43; *p* = 0.52, partial η^2^ = 0.02] and no interaction effect [*F*(1, 21) = 0.35; *p* = 0.60, partial η^2^ = 0.02] on the task accuracy were found. Regarding the N400 priming effect, a main effect of the writing tool was found [*F*(1, 21) = 11.05; *p* = 0.003, partial η^2^ = 0.35] as the priming effect was larger for words written with a digital pen than those written with an ink pen. There was no significant main effect of the experiment [*F*(1, 21) = 2.37; *p* = 0.14, partial η^2^ = 0.10] and no interaction effect [*F*(1, 21) = 0.02; *p* = 0.88, partial η^2^ = 0.001].

In the unfamiliar group (Figure 6B), a significant main effect of the experiment was found on the number of memorized words [*F*(1, 36) = 5.21; *p* = 0.03, partial η^2^ = 0.13] and task accuracy [*F*(1, 36) = 9.54; *p* = 0.004, partial η^2^ = 0.21]. In addition, the main effect of the writing tool on the number of memorized words was significant [*F*(1, 36) = 7.21; *p* = 0.01, partial η^2^ = 0.17], but no significant interaction effect was detected [*F*(1, 36) = 0.15; *p* = 0.70, partial η^2^ = 0.004]. This indicates that the performance in the post-learning test was lower in the digital pen condition compared with the ink pen condition. There was no significant main effect of the writing tool [*F*(1, 36) = 2.35; *p* = 0.13, partial η^2^ = 0.06] and no interaction effect [*F*(1, 36) = 0.01; *p* = 0.91, partial η^2^ = 0.0004] on the task accuracy. A marginal effect of the experiment [*F*(1, 36) = 3.88; *p* = 0.057, partial η*^2^* = 0.10] and interaction effect between the experiment and writing tool [*F*(1, 36) = 2.83; *p* = 0.10, partial η^2^ = 0.07] were found on the N400 priming effect. This indicates that the N400 priming effect in the digital pen condition in the present study was significantly smaller than in the previous study [*t*(36) = 2.78, *p* = 0.009], whereas the priming effect under the ink pen condition was not significantly different between the experiments [*t*(36) = 0.42, *p* = 0.67].

## Discussion

In this study, participants learned words from a foreign language using three different writing tools: handwriting with a pen on paper, handwriting with a digital pen on a tablet, and typing on a keyboard. To our knowledge, this study is the first to provide a complete analysis of multiple factors that influence learning of words. Indeed, we not only measured the impact of the tool on the learning process, taking into account the familiarity with the digital tools, but also directly compared the mood states during learning. Moreover, behavioral and electroencephalographic indices were measured immediately after learning and were further compared between participants who were familiar with writing using a digital pen and those who were not. Regarding the behavioral indices, we showed that the number of writing repetitions per word was greater when typing than it was when handwriting with an ink pen and a digital pen. However, the performances at the post-learning test and judgment task in the EEG experiment were not affected by the writing tools. The repetition priming effect on N400 was larger for words learned by handwriting, regardless of the pen type, than those learned by typing. Furthermore, a significant interaction between the participant groups and writing tools was found. Interestingly, the familiar group showed a greater N400 priming effect for words learned with the digital pen or ink pen than those learned using the keyboard. However, in the unfamiliar group, this greater N400 priming effect concerned only words learned with the ink pen. In addition, we also show that an index of positive mood was higher when handwriting with a pen on paper, compared with typing.

Handwriting is more efficient in learning than typing only considering the N400 priming, but not the performance level. This might reflect a difference in the sensitivity for early stage learning between the EEG and behavioral indices. Indeed, an ERP study by McLaughlin et al. (2004) reported a difference in N400 amplitude between words from a second language (L2) compared with pseudo-words after only 14 h of classroom instruction, while it took longer to observe a difference in the performance in the lexical decision task. This finding suggests that the amplitude modulation of N400 is more sensitive and reflects a stage of language learning compared earlier than is evident in the behavioral indices. In another study where adult participants learned new characters either by handwriting or typing for 3 weeks (1 h/week), no difference in the rate of letter recognition accuracy was evidenced immediately after the end of the training session. However, the rate of recognition accuracy for the typed characters, but not the handwritten one, gradually decreased over the 3 weeks following the training (Longcamp et al., 2006). This constitutes another clear example where the advantage provided by handwriting over typing at the behavioral level was not revealed immediately after training. A similar conclusion was also drawn from another recent study comparing alphabet training in preschool children for 7 weeks by handwriting with a pencil on a sheet of paper, handwriting with a stylus on a tablet computer, or typing letters using a virtual keyboard on a tablet (Mayer et al., 2020). Indeed, no difference regarding letter recognition was evidenced immediately after the training, but a clear advantage of handwriting with a pencil over typing was shown at a follow-up assessment about 4–5 weeks after the training. It should also be noted that the training period in these studies was considerably longer (more than a few weeks) than in the present study (10 min), although there was no difference in the learning content and assessment methods. Our results suggest that the ERP is effective for detecting an effect on learning after a short period of use.

Although handwriting, regardless of the pen type, showed a superior learning effect (i.e., a larger N400 priming effect), the number of writing repetitions per word was higher with typing. This indicates that typing provides the advantage of allowing to write more words; however, this does not contribute to test performance. Similar results have been reported in a behavioral study by Mueller and Oppenheimer (2014). In their study, university students took notes by handwriting or typing while listening to the lecture. They then took a test containing factual and conceptual questions. Students who typed notes wrote more words than those who handwrote notes; however, they performed worse on the conceptual questions. It was proposed to result from the fact that students who type tend to transcribe lectures verbatim rather than process the information and rephrase it in their own words. In our study, the participants were required to copy the Indonesian words presented on the learning sheets; therefore, the effect of the above factor can be excluded. Previous behavioral studies argued that the learning advantage of handwriting over typing is due to the motor-perception integration occurring during handwriting as handwriting movements facilitate the recognition of abstract graphic forms (Hulme, 1979; Naka and Naoi, 1995), letters (Longcamp et al., 2005, 2006), and written words (Kiefer et al., 2015). This hypothesis has been supported by MRI (Longcamp et al., 2008) and EEG studies (Ose Askvik et al., 2020). In the MRI study, a greater activity in response to letters learned by handwriting, compared with those learned by typewriting, was observed in several brain regions involved in the execution, imagery, and observation of actions, such as the left Broca’s area and bilateral inferior parietal lobules. In the EEG study, event-related theta-band synchronization in the parietal and central regions was observed when the participants were writing with a digital pen on a touchscreen, whereas event-related theta- and alpha-band desynchronization in the alpha range were found when the participants were typewriting on a keyboard. In line with these studies, the present results suggest that handwriting movements, regardless of the pen type, allowed a better memorization of new words compared with typing which provided the advantage of writing more words.

We propose that a more positive mood during training might also explain the higher learning efficiency of handwriting compared with typing. Indeed, using the POMS, we show that the participants felt more *Vigor or Activity* when handwriting with an ink pen than when typing, whereas negative moods, such as *Tired and Tension*, were not affected by the writing tool. Previous ERP studies showed that mood affects language comprehension (Federmeier et al., 2001; Vissers et al., 2010, 2013; Chwilla et al., 2011; Pinheiro et al., 2013) and production (Hinojosa et al., 2017). According to these studies, positive mood can facilitate semantic processing. For example, Federmeier et al. (2001) pointed out that mild, transient positive mood leads to access a richer set of semantic properties for upcoming words in a sentence. Therefore, we speculate that the increased positive mood reported while handwriting with an ink pen may facilitate semantic access to the words to be learned. As a result, more semantic representations might be activated for words that were handwritten in the post-learning priming experiment, and a greater priming effect on N400 was produced.

A significant interaction effect on the N400 repetition priming effect was obtained for writing tool by participant group. In the familiar group, the learning effect in the digital pen condition was significantly greater than it was in the keyboard condition and with trend for greater than the effect in the ink pen condition. In the unfamiliar group, the learning effect in the ink pen was non-significant but showed a trend to be greater than the effect in the keyboard condition. These results suggest that handwriting with a digital pen provides an advantage over typing for those familiar with using digital pens, while writing with ink pens might be more advantageous for those unfamiliar with digital tools. In addition, the comparison of the present results with those from our previous study (Osugi et al., 2019) suggests that the familiarity with the writing tools and the difficulty of the learning task influence the learning capacity. Here participants had to write and memorize words from an unknown foreign language (Indonesian) while also writing and memorizing readings of word from their native language (Japanese) in the 2019 study. Regardless of the group, the subjects performed worse for all behavioral indices (i.e., number of memorized words in the post-learning test and task accuracy during the ERP experiment) in the present study. This suggests that the learning task was more difficult in this study than in the previous one. In the familiar group, the difficulty of the learning task had no significant effect on the N400 priming effect. However, in the unfamiliar group, the N400 priming effect for the digital pen condition was significantly smaller in the present study than in the previous studies, while there was no significant difference for the ink pen condition. These results suggest that the difficulty of the task affects learning ability using a digital pen of those who are not accustomed to it. In a behavioral study, Mayer et al. (2020) compared the alphabet learning capacity in preschool children trained by handwriting with a pencil, handwriting with a stylus, and typing on a keyboard during 7 weeks. They showed that handwriting with a pencil improved the performance in letter knowledge and visuospatial skills compared with keyboarding. In contrast, training using a stylus was less efficient for word reading and writing than training using the keyboard. They proposed that writing with a stylus on a touchscreen is the least favorable writing tool probably because of the higher need of motor control. Our results from the familiar group provide evidence that writing with a digital pen and tablet is a better learning tool than typing once individuals are accustomed to it.

There are some limitations to this study. First, although the two-way ANOVA examining the N400 priming effect clearly showed a significant main effect of the writing tool, with a greater effect for handwriting than for typing, the *post hoc* analysis divided by group showed that the difference between handwriting and typing was not significant in the unfamiliar group (*p* = 0.06). Based on the results of a *post hoc* power analysis on the one-way ANOVA (power = 0.62), we suggest that this may be due to the small sample size (*n* = 21). Second, we investigated the difference in behavioral and electroencephalographic indices measured immediately after learning. It remains to be determined if the difference in the repetition priming effect on N400 between handwriting and typing persists in the long term. Third, there is a possible confound, in that five of the subjects also participated in our previous study (Osugi et al., 2019) and thus had some experience in the type of learning and testing phases used. Fourth, we selected participants for the familiar group based on the frequency (i.e., more than a few days a week), but not the duration of use, which might also affect the learning capacities. Finally, we did not measure the time that the participants’ spent monitoring each word they were writing or examine the relationship between this and the type of writing tool used. The difference in the time their working memories spent monitoring each word may have played a role in the observed difference in the learning effects. This possibility should be clarified by future experiments.

To our knowledge, this work is the most complete study so far regarding the impact of handwriting with a pen, a digital pen, or a keyboard on learning processes. Indeed, we have taken into account the familiarity with the tools used to learn and mood state of the participants during training. This works opens new paths of research to better understand the learning processes and improve the efficiency of digital education, which is increasing in demand in the current educational environment.

## Data Availability Statement

The data that support the findings of this study are available upon request from the corresponding author.

## Ethics Statement

The studies involving human participants were reviewed and approved by the Bioinformatics Ethics Committee of the National Institute of Information and Communications Technology. The participants provided their written informed consent to participate in this study.

## Author Contributions

AI, YN, KN, AK, and KI designed the study. KN, KO, and KI conducted the experiments. KN prepared the materials and experimental equipment, and analyzed the data. AI wrote the first draft of the manuscript. All authors revised the draft and approved the final version.

## Conflict of Interest

AK and KI were employed by Wacom Co., Ltd. A part of this study was conducted with a research fund from Wacom Co., Ltd. The funder had the following involvement with the study: study design and collection. The remaining authors declare that the research was conducted in the absence of any commercial or financial relationships that could be construed as a potential conflict of interest.

## Footnotes

1 https://pj.ninjal.ac.jp/corpus_center/bccwj/en/

2 http://psychtoolbox.org/
