# Supplementary material for: Advantage of Handwriting Over Typing on Learning Words: Evidence From an N400 Event-Related Potential Index
Source: Front Hum Neurosci. 2021 Jun 10;15:679191. doi: 10.3389/fnhum.2021.679191 (PMC8222525; doi:10.3389/fnhum.2021.679191)
Supplement: Supplementary file 1 [file Data_Sheet_1.docx]

Supplementary Materials

**Supplementary Table 1.** Indonesian words learned.

| **Set 1** | | **Set 2** | | **Set 3** | |
| --- | --- | --- | --- | --- | --- |
| Indonesian | English translation | Indonesian | English translation | Indonesian | English translation |
| Kanan | Right | Guru | Teacher | Malam | Night |
| Kiri | Left | Anak | Children | Sore | Evening |
| Hitam | Black | Teman | Friend | Siang | Noon |
| Putih | White | Ayah | Father | Pagi | Morning |
| Biru | Blue | Ibu | Mother | Bahu | Shoulder |
| Merah | Red | Gajah | Elephant | Jari | Finger |
| Hijau | Green | Sapi | Cow | Kuku | Nail |
| Pohon | Tree | Rusa | Deer | Wajah | Face |
| Laut | Sea | Ular | Snake | Perut | Stomach |
| Tanah | Ground | Babi | Pig | Gigi | Tooth |
| Hutan | Woods | Buaya | Crocodile | Lidah | Tongue |
| Danau | Lake | Domba | Sheep | Dada | Breast |
| Kolam | Pond | Macan | Tiger | Leher | Neck |
| Pulau | Island | Kuda | Horse | Siku | Elbow |
| Gula | Sugar | Jam | Clock | Mata | Eye |
| Susu | Milk | Koran | Newspaper | Lutut | Knee |
| Udang | Shrimp | Tas | Bag | Bibir | Lips |
| Ikan | Fish | Meja | Desk | Pipi | Cheek |
| Roti | Bread | Kursi | Chair | Mulut | Mouth |
| Sayur | Vegetables | Pintu | Door | Kaki | Leg |


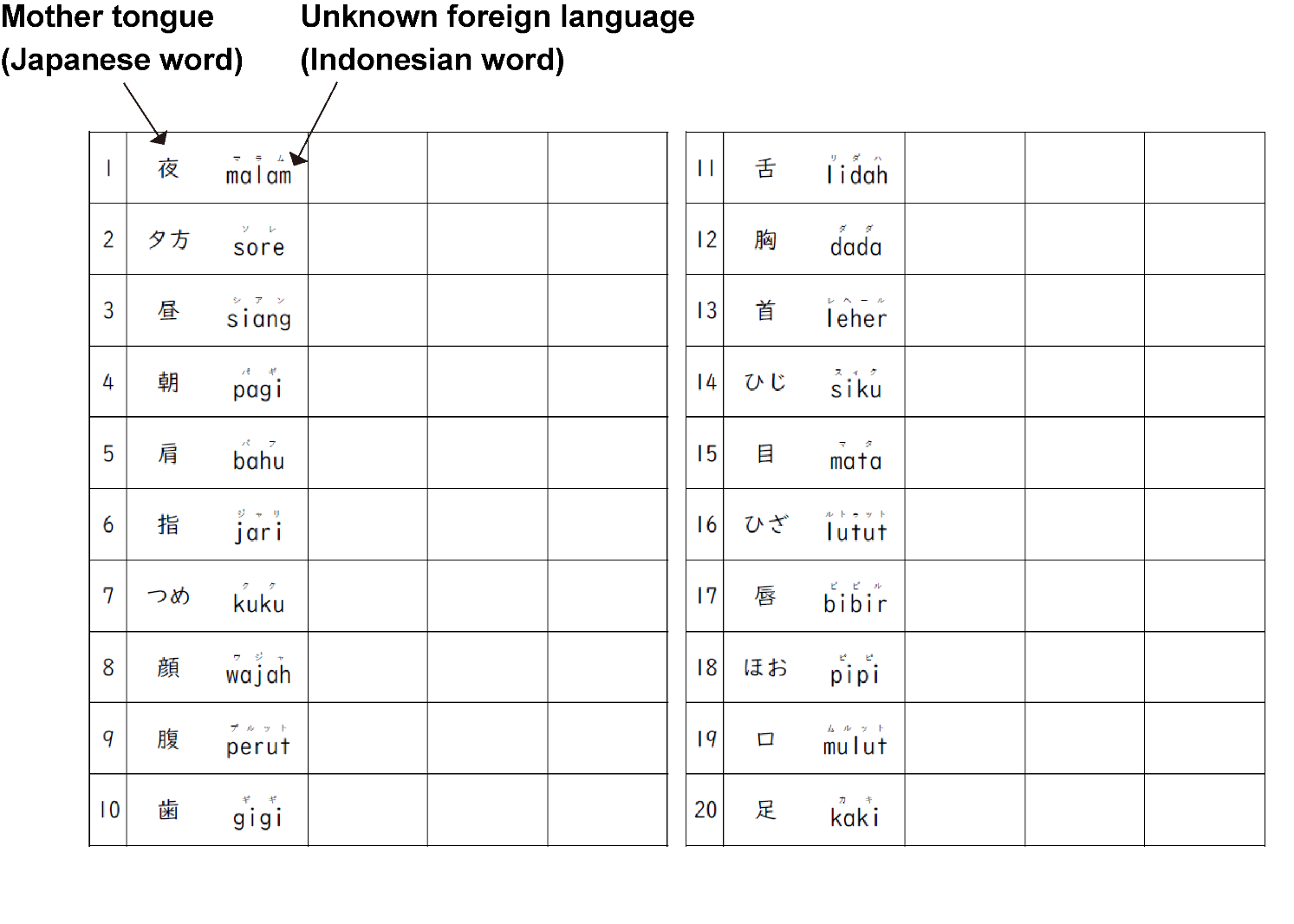


**Supplementary Figure 1. Example of a learning sheet used in the learning activity sessions.** Each sheet contained 20 Indonesian words and their Japanese translations, and the participants were asked to write the Indonesian words repeatedly in the empty spaces provided, in order to try to memorize them. A paper version was provided in the ink pen condition, and an electronic version was provided for the digital pen and keyboard conditions, on a tablet and a PC monitor, respectively.


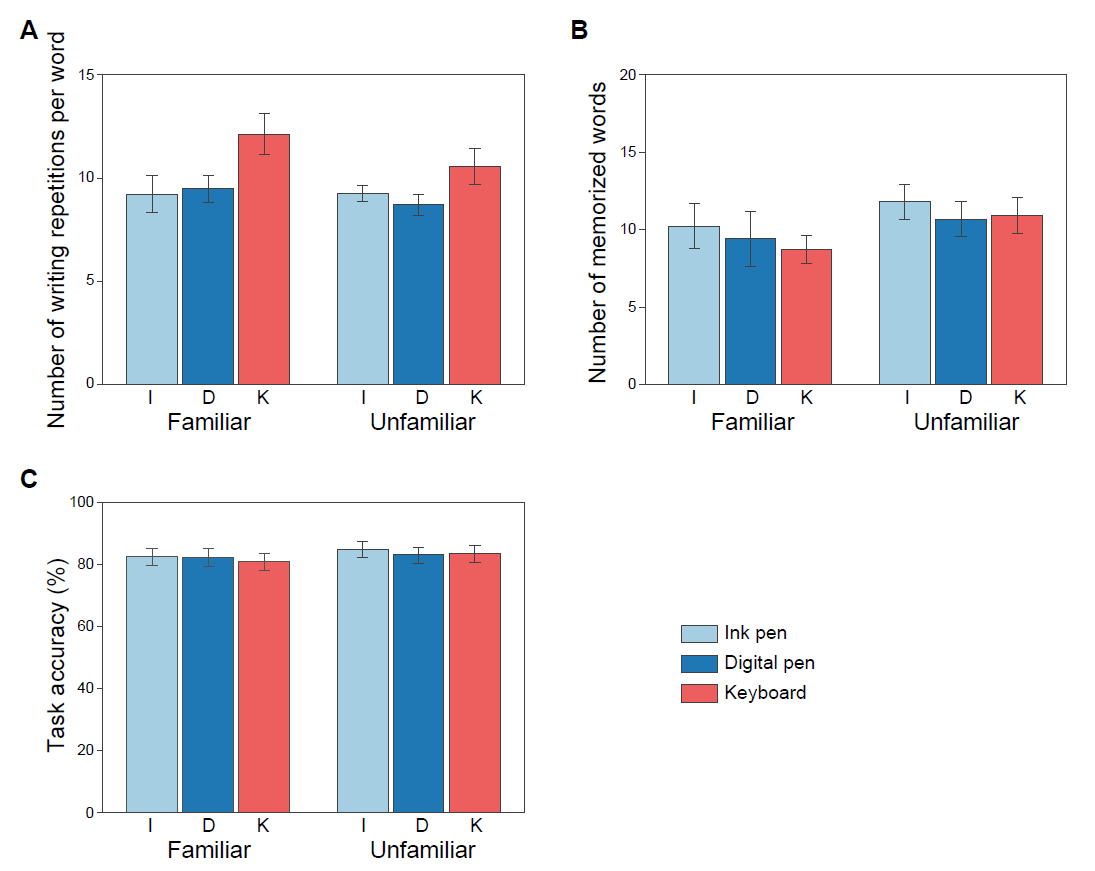


**Supplementary Figure 2. Learning effect on behaviors in the familiar and unfamiliar groups.** The number of writing repetitions per word during the learning activity (A), numbers of memorized words in the post-learning test (B), and task accuracy in the EEG experiment (C) showed no main effect of the group and no interaction effect between the group and learning method. Each bar shows the grand average of the participants. The error bar represents the standard error.

**
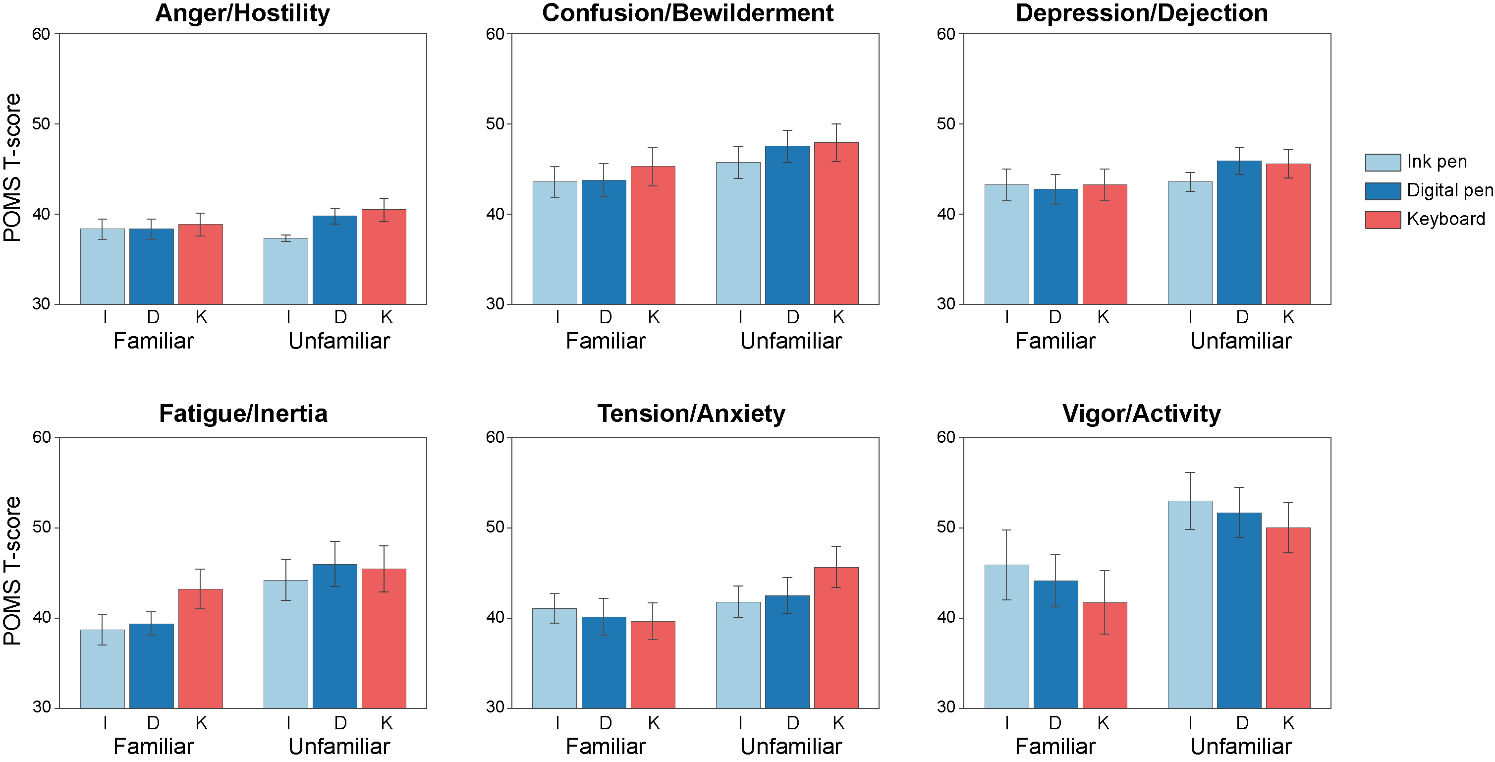
**

**Supplementary Figure 3. POM scores in the familiar and unfamiliar groups.** The POMS T-scores showed no main effect of group and no interaction effect between the group and learning method. Each bar shows the grand average of the participants. The error bar represents the standard error.
